# Predictors of therapy failure in newly diagnosed pulmonary tuberculosis cases in Beira, Mozambique

**DOI:** 10.1186/s13104-018-3209-9

**Published:** 2018-02-05

**Authors:** Damiano Pizzol, Nicola Veronese, Claudia Marotta, Francesco Di Gennaro, Jorge Moiane, Kajal Chhaganlal, Laura Monno, Giovanni Putoto, Walter Mazzucco, Annalisa Saracino

**Affiliations:** 1 Research Unit, Doctors with Africa-CUAMM, Beira, Mozambique; 2 Department of Medicine (DIMED)-Geriatrics Section, University of Padova, Padua, Italy; 3 Department of Science for Health Promotion and Mother to Child Care “G. D’Alessandro”, University of Palermo, via del Vespro, 133, Palermo, Italy; 4 Department of Infectious Diseases, University of Bari “Aldo Moro”, P.zza G Cesare 3, Bari, Italy; 5 Doctors with Africa-CUAMM, Padua, Italy; 6 Faculty of Health Sciences, Catholic University of Mozambique, Center for Research in Infectious Diseases, Beira, Mozambique; 7 Research Section, Doctors with Africa CUAMM, Padua, Italy

**Keywords:** Tuberculosis, Mozambique, Therapy failure

## Abstract

**Objective:**

Tuberculosis (TB) remains a major global health issue, ranking in the top ten causes of death worldwide. A deep understanding of factors influencing poor treatment outcomes may allow the development of additional treatment strategies, focused on the most vulnerable groups. Aims of the study were: (i) to evaluate the treatment outcome among TB subjects followed in an outpatient setting and (ii) to analyze factors associated with treatment failure in newly diagnosed patients with pulmonary TB in Beira, the second largest city of Mozambique.

**Results:**

A total of 301 TB adult patients (32.6% females) were enrolled. Among them, 62 (20.6%) experienced a treatment failure over a 6 months follow-up. On multivariate model, being males (O.R. = 1.73; 95% CI 1.28–2.15), absence of education (O.R. = 1.85; 95% CI 1.02–2.95), monthly income under 50 dollars (O.R. = 1.74; 95% CI 1.24–2.21) and being employed (O.R. = 1.57; 95% CI 1.21–1.70), low body mass index values (O.R. = 1.42; 95% CI 1.18–1.72) and HIV status (O.R. = 1.42; 95% CI 1.10–1.78) increased the likelihood of therapy failure over 6 months of follow-up. In this study, patients who need more medical attention were young males, malnourished, with low income and low educational degree and HIV positive. These subjects were more likely to fail therapy.

## Introduction

Tuberculosis (TB) remains a major global health issue, ranking in the top ten causes of death worldwide [1]. In 2015, 10.4 million people were estimated as newly diagnosed with TB and 1.8 million deaths were registered worldwide [1], not equally distributed all over the world. Particularly, over 90% of global TB cases and deaths occurs in low and middle income countries, and especially in fragile states [2]. Moreover, despite effective anti-tuberculosis chemotherapy, case-fatality rates of up to 25% are described in both industrialized and resource-poor settings [3].

Mozambique is ranked 19th among the 22 TB High Burden Countries in the world, with an estimated TB incidence rate of 551/100.000 population in 2015 [4]. However, TB treatment covers only 38% of the population, and an increasing rate of TB–HIV co-infection is usually documented (49% of TB patients were HIV-positive) [4]. In Mozambique, the treatment success rate among new sputum smear-positive cases has increased from 76% in 2003–79% in 2007 and 87% in 2015 [4].

In this African Country, among the many challenges dealing with TB control, both ensuring proper TB diagnosis and treatment as well as implementing the one-stop model for TB care have been regarded as national priorities [5] Worryingly, recent data from Manhiça, a district located in southern Mozambique, show an alarming mortality rate among TB adult cases co-infected with HIV [6]. The potential threat of increasing multi-drug resistance (MDR) cases in the country—the latest national survey documented a MDR incidence of about 3.5% among new TB cases—could jeopardize the achievement of the treatment success targets set in the strategic plan 2014–2018 of the National TB Control Programme (NCTP) [5, 7].

A deep comprehension of factors influencing poor treatment outcome may allow the development of additional treatment strategies, focusing on the most vulnerable groups. Unfortunately, very few data are available addressing this issue, particularly in Mozambique. In general, predictors for unsuccessful treatment are considered socio-demographic, behavioral, disease-related and treatment-related factors [8]. In Mozambique, only one study conducted in the country highlighted factors associated with a higher risk of death during the TB treatment: HIV status, being male and lack of laboratory confirmation [6]. For these reasons, the aims of our study were (i) to evaluate the treatment outcomes among TB subjects followed in an outpatient setting and (ii) to analyze factors associated with treatment failure in newly diagnosed cases with pulmonary TB in Beira, the second largest city of Mozambique.

## Main text

### Study population and design

An observational study was implemented.

All cases of pulmonary TB diagnosed between January and August 2016 were recruited in this observational study from three urban outpatient health-care centers of Beira district, namely Ponta-Gea, Munhava and Macurungo, the largest in Sofala’s Province, involved in the National Tuberculosis Control Programme (NCTP).

Inclusion criteria were to be a subject aged ≥ 18 years, having a confirmed TB diagnosis (positive sputum smear result, GeneXpert positive result, culture positive result or Positive chest X-ray) [1] and able to undergo anti-TB treatment, not having any anti-TB treatment or TB treatment started within the previous 2 months.

Anti-TB treatment was prescribed according to the Mozambique National Tuberculosis and Leprosy Control Programme guidelines [5]. So, when GeneXpert was negative for resistances, patients underwent an initial phase of therapy with Isoniazid, Rifampin, Pyrazinamide and Ethambutol lasting the first 2 months and then a continuation phase with Isoniazid and Rifampin for the next fourth months. While, when GeneXpert documented a resistance to Rifampin a second line therapy was implemented.

At the end of therapy every patient underwent a general visit, X-rays and/or sputum smear, and diabetes diagnosis test.

A face-to-face interview conducted by a trained nurse encompassed questions about demographic characteristics (age, residence, education, occupation, marital status, monthly income), pregnancy, behaviours (sexual behaviour, concurrent sex partners, condom use, smoke and alcohol abuse, etc.) and medical history, including TB and diabetes symptoms. A basic physical examination (vital signs, weight, height, waist circumference, blood pressure and general appearance) was performed. The body mass index (BMI) was calculated. Moreover, subjects with unknown HIV status received a pre-HIV test or a rapid HIV test, if not done before.

Each participant, during two consecutive clinic visits, underwent two fasting blood glucose tests to investigate on diabetes: according to WHO guidelines, patients were considered as non-diabetic if both measurements were ≤ 110 mg/dl, and as diabetic if both measurement were above 126 mg/dl. If at least one value was between 110 and 126 mg/dl, the Oral Glucose Tolerance Test (OGTT) was further performed: patients were considered diabetic when plasma glucose at 2 h was ≥ 200 mg/dl [9]. All TB-diabetic patients underwent assessment of diabetic complications.

Treatment outcomes were defined according to WHO criteria [10]. Successful treatment outcome was defined as a clinical and radiological improvement in a patient with a baseline smear positivity and evidence of at least two negative sputum smears, the first during the maintenance period, and the second as the treatment was completed. Treatment failure was defined as the detection of positive sputum smear in a patient at month 5 or later of treatment.

### Statistical analysis

Data were reported as mean and standard deviations for continuous variables. Absolute and relative frequencies (percentages) were used for categorical variables.

Independent T-test was used to compare groups for continuous variables, whilst a Chi square test (with the Fisher’s correction if less than 5 cases were present in a cell) was applied for categorical variables.

A logistic regression model was implemented as follows. Treatment failure was considered as dependent variable and each one of the available factors at the baseline evaluation as independent variables (univariate analysis). In the multivariate analysis all the factors with a p-value < 0.10 at the univariate analyses were included. Multicollinearity among covariates was assessed through the variance inflation factor (VIF), taking a value of 2 for excluding a covariate. However, no variable was excluded according to the previous criterion.

Odds Ratios (ORs) as adjusted Odds Ratios (Adj–ORs) with their 95% confidence intervals (CIs) were used to measure the association between factors at the baseline (exposure) and treatment failure (outcome).

All statistical tests were two-tailed and statistical significance was assumed for a p-value < 0.05. Analyses were performed by using the SPSS 21.0 for Windows (SPSS Inc., Chicago, Illinois).

## Results

A total of 301 TB adult patients (32.6% females) were enrolled in the study (Table 1), distributed among the three outpatient health-care centers as follow: 132 (43.8%) Ponta-Gea, 63 (20.9%) Munhava and 106 (35.2%) Macurungo. The whole sample had a mean age of 31 years (S.D.: 12.5). Nearly half of the participants (48.2%) reported to have no education, 187 (62.0%) participants were employed and only 83 (27.6%) had a monthly income higher than 50 dollars.

**Table 1:** Characteristics of the 301 recruited TB patients and differences in treatment outcomes

Characteristics	Study population n. 301	Treatment		p-value
		Failure n. 62	Success n. 239	
Average age (years)	31 (12.5)	33.3 (12.8)	30.4 (12.1)	0.84
Female n. (%)	98 (32.6)	24 (24.5)	74 (75.5)	< 0.0001
No education	145 (48.2)	41 (28.3)	104 (71.7)	< 0.0001
Employed	187 (62.0)	13 (6.9)	174 (93.1)	< 0.0001
Monthly income ≤ 50 dollars	218 (72.4)	49 (22.5)	169 (77.5)	< 0.0001
Lifestyles and behaviours				
Current smoking	25 (8.3)	17 (68.0)	8 (32.0)	< 0.0001
Alcohol use	38 (12.6)	21 (55.3)	17 (7.1)	<0.0001
Sexual partner/s				< 0.0001
No	110 (36.5)	24 (21.8)	86 (78.2)	< 0.0001
Yes	184 (61.1)	35 (56.4)	149 (62.3)	
Medical history and examination				
Low BMI (< 18)	169 (56.0)	45 (26.6)	124 (73.4)	< 0.0001
HIV positive	131 (43.5)	39 (37.4)	92 (62.6)	< 0.0001
Antiretroviral treatment (ART)	93 (30.9)	18 (19.3)	75 (80.7)	< 0.0001
Diabetes or IGT	9 (3.0)	6 (66.7)	3 (33.3)	< 0.0001
Positive chest X-ray	148 (49.2)	9 (6.0)	139 (94.0)	< 0.0001
Positive sputum examination	246 (81.7)	40 (16.3)	206 (83.7)	< 0.0001
Positive GeneXpert	56 (18.6)	35 (62.5)	21 (37.5)	< 0.0001
Symptoms before treatment				
Cough	296 (98.3)	59 (20.4)	237 (79.6)	<0.0001
Loss of weight	261 (86.7)	33 (12.6)	228 (87.4)	< 0.0001
Astenia	215 (71.4)	25 (11.6)	190 (88.4)	< 0.0001
Fever	174 (57.8)	24 (13.8)	150 (86.2)	< 0.0001
Night sweat	202 (67.1)	26 (12.8)	176 (87.2)	< 0.0001

With regard to participants’ lifestyle and behaviour information, 25 participants (8.3%) reported to be current smokers and 38 (12.6%) daily drinkers. The majority of the participants reported to have one sexual partner, whereas only 7 (4.7%) reported to have more than one, and the remaining part (36.5%) had no partner.

According to medical history and examination, 56.0% of participants presented a BMI (< 18.5) below the healthy range, whereas arterial hypertension was found just in only 1 patient (0.3%).

The HIV status was known to be positive in 131 patients (43.5%) and 93 (70.9%) of them were receiving antiretroviral treatment (ART). Furthermore, diabetes or impaired glucose tolerance (IGT) was diagnosed in 9 patients (3%).

TB was diagnosed by a positive chest X-ray examination in 148 patients (49.2%); 246 patients (81.7%) had a positive sputum examination and 56 patients (18.6%) a positive GeneXpert sputum test.

The most reported symptoms before the initiation of treatment were cough (98.3%), loss of weight (86.7%), asthenia (71.4%), night sweats (67.1%) and fever (57.8%).

A total of 62 patients (20.6%) experienced a treatment failure over a 6-month follow-up.

No difference was documented between patients experiencing treatment failure or success as regards age (p-value: 0.84) and gender (p-value: 0.25). On the contrary, employed subjects (93.1%; p-value < 0.0001), with no education (71.7%; p-value < 0.0001) and with a monthly income < 50 dollars (77.5%; p-value < 0.0001) were more frequent in the success group, whereas daily alcohol users (55.3%; p-value < 0.0001) and current smokers (77.5%; p-value < 0.0001) exceeded in failure one.

The multivariate model considered the effects of gender, BMI, education, alcohol use, being employed, monthly income, smoking habits, HIV status, being on ART, sputum examination and GeneXpert positivity. Significant predictors of therapy failure over 6 months of follow-up are reported in Table 2: male sex (O.R. = 1.73; 95% CI 1.28–2.15), absence of education (O.R. = 1.85; 95% CI 1.02–2.95), monthly income under 50 dollars (O.R. = 1.74; 95% CI 1.24–2.21) and being employed (O.R. = 1.57; 95% CI 1.21–1.70), low BMI values (O.R. = 1.42; 95% CI 1.18–1.72) and HIV status (O.R. = 1.42; 95% CI 1.10–1.78).

**Table 2:** Predictors of treatment failure over 6 months of follow-up

Characteristics	Univariate analysis O.R.	Multivariate analysis Adj–O.R.
Age (years)	1.02 (0.98–1.04)	–
Female	0.28 (0.16–0.40)	0.58 (0.47–0.78)*
Low BMI (< 18)	1.80 (1.42–2.02)	1.42 (1.18–1.72)*
No education	1.50 (1.28–1.74)	1.85 (1.02–2.95)*
Alcohol use	0.04 (0.00–0.10)	0.45 (0.08–1.03)
Employed	0.51 (0.43–0.70)	0.64 (0.59–0.83)*
Monthly income ≤ 50 dollars	1.85 (1.35–2.45)	1.74 (1.24–2.21)*
Actual smoking	2.02 (1.27–2.53)	1.33 (0.85–178)
Sexual partner/s	0.10 (0.04–0.85)	0.39 (0.08–1.28)
Positive chest X-ray	–	–
HIV positive	1.80 (1.50–2.00)	1.42 (1.10–1.78)*
Antiretroviral treatment (ART)	0.64 (0.38–0.78)	0.74 (0.50–1.03)
Diabetes or IGT	–	–
Positive sputum examination	0.25 (0.10–0.40)	0.71 (0.28–1.23)
Positive GeneXpert	2.00 (1.45–2.55)	1.28 (0.74–2.79)

* p < 0.05

## Limitations

Very few data are available about the outcome of TB treatment in high burden countries, leaving a lack of important evidences for the implementation of future public health and clinical strategies. In our sample of patients referring to the three urban health centers of the Beira District, a percentage of 20% of patients experiencing a treatment failure over a 6 months follow-up was found.

In the present study these unsuccessful treatment outcomes were found to be associated with a low education level, low income and low BMI (< 18.5), resulting as the most relevant predictors of therapy failure from the multivariate analysis.

In fact, in our sample nearly half of patients had no educational degree and almost 70% had a monthly income lower than 50 dollars, defining a low socioeconomic status. This patients’ profile, according to the literature [11, 12], is more likely to have less awareness of health issues, reduced access to health services, low self-care attention, resulting in delays in the TB diagnosis and treatment. Also, patients with low education level will be more likely to misuse and discontinue drug use during treatment [13, 14].

Similarly more then half of our patients presented a BMI below the healthy range, and it is well known that malnutrition is associated with an increased risk of mortality and relapse of active TB, whereas the use of macronutrient supplementation during treatment with weight gain at 2 months may result in improvement in physical function, sputum conversion and treatment completion [15].

In agreement with current literature, in our study, an association of poor TB treatment outcome with HIV infection was found [16, 17]. HIV co-infection is the most important risk factor for developing active TB, which increases susceptibility to primary infection, re-infection and/or reactivation of latent TB. TB also has a negative impact on the immune response to HIV, increasing the progression from HIV infection to acquired immunodeficiency syndrome (AIDS) [18].

The risk of poor treatment outcome in that particular subpopulation is higher. Hence, a particular attention should be destined for HIV/TB-co-infected patients whose TB treatment is burdened by frequency of drug administration, pill burden, drug interactions, drug toxicity and worse general health conditions, [19, 20].

Instead, we did not find additional risk factors for TB treatment default such as smoking and alcohol use [21], probably because our data could be underreported because they were not verified and were based on personal interview.

This study has some limitations. First, no sufficient data on MDR or XDR, no data on check at 2 month from start therapy and delay in diagnosis. This points, together with a deeper exploration of the role of comorbidities, such as diabetes [22] should influence future research on this field. On the contrary strengths are the high number of patients and the data from country where there are few notice about treatment outcome.

We describe the phenotype of patients that need more medical attention: young, male, malnourished, with low income and low educational degree, HIV positive. They have more possibility to failure therapy and they need close follow up. Counseling and education on TB is always recommended in this more vulnerable patients [23, 24].

These considerations suggest that a drug approach is not enough to treat tuberculosis and further policies will have to take into account a multidisciplinary approach including education and social equity. Moreover, it is crucial to create a simple and effectiveness monitoring system, allowing a national overview also on multi-drug resistance and delay in diagnosis, in order to obtain complete and quality data to guide public health actions.

In conclusion, this study underlines that only pharmacological approach isn’t longer sufficient to guarantee a reduction of TB burden. Social determinants of health have a crucial role, and more work, more instruction, in other words less poverty, HIV educational associated with pharmacological approach and early diagnosis can real improve TB patients outcomes and global TB burden. We believe that this is the way forward.

## Abbreviations

TB: tuberculosis
WHO: World Health Organization
OR: odds ratio
BMI: body mass index
HIV: human immunodeficiency virus
MDR: multi-drug resistance
NCTP: National Tuberculosis Control Programme
OGTT: Oral Glucose Tolerance Test
VIF: variance inflaction factor
Adj–ORs: adjusted odds ratios
ART: antiretroviral treatment
IGT: impaired glucose tolerance
SDH: social determinants of health
